# Population Connectivity Shifts at High Frequency within an Open-Coast Marine Protected Area Network

**DOI:** 10.1371/journal.pone.0103654

**Published:** 2014-07-31

**Authors:** Geoffrey S. Cook, P. Ed Parnell, Lisa A. Levin

**Affiliations:** 1 Cooperative Institute for Marine and Atmospheric Studies, Rosenstiel School of Marine and Atmospheric Science, University of Miami, Miami, FL, United States of America; 2 Atlantic Oceanographic and Meteorological Laboratory, National Oceanographic and Atmospheric Administration, Miami, FL, United States of America; 3 Integrative Oceanography Division, Scripps Institution of Oceanography, University of California San Diego, La Jolla, CA, United States of America; The Australian National University, Australia

## Abstract

A complete understanding of population connectivity via larval dispersal is of great value to the effective design and management of marine protected areas (MPA). However empirical estimates of larval dispersal distance, self-recruitment, and within season variability of population connectivity patterns and their influence on metapopulation structure remain rare. We used high-resolution otolith microchemistry data from the temperate reef fish *Hypsypops rubicundus* to explore biweekly, seasonal, and annual connectivity patterns in an open-coast MPA network. The three MPAs, spanning 46 km along the southern California coastline were connected by larval dispersal, but the magnitude and direction of connections reversed between 2008 and 2009. Self-recruitment, i.e. spawning, dispersal, and settlement to the same location, was observed at two locations, one of which is a MPA. Self-recruitment to this MPA ranged from 50–84%; within the entire 60 km study region, self-recruitment accounted for 45% of all individuals settling to study reefs. On biweekly time scales we observed directional variability in alongshore current data and larval dispersal trajectories; if viewed in isolation these data suggest the system behaves as a source-sink metapopulation. However aggregate biweekly data over two years reveal a reef network in which *H. rubicundus* behaves more like a well-mixed metapopulation. As one of the few empirical studies of population connectivity within a temperate open coast reef network, this work can inform the MPA design process, implementation of ecosystem based management plans, and facilitate conservation decisions.

## Introduction

Accurate and robust estimates of larval dispersal and local retention are essential prerequisites to resolving marine colonization patterns, controlling the spread of invasive species, and the design of effective marine protected areas (MPAs) [1]–[4]. Ecological benefits exist within and outside the boundaries of MPAs [5]–[7]. Within MPAs these benefits include increases in abundance, diversity, and productivity of marine communities; lower levels of mortality within MPAs decreases the probability of local and regional extinction [6], [8]. Outside of MPAs, benefits accrue when individuals move beyond MPA boundaries (i.e. spillover) increasing the biomass of catchable fish in waters adjacent to MPAs. In addition MPAs may sustain populations on a regional scale by larval dispersal and adult/juvenile movement among reefs [9]. Documentation of MPA self-recruitment and connectivity via larval dispersal will aid in the assessment of MPA success [4]. It will also advance our understanding of the benefits MPAs provide marine natural resources yielding useful information for managers tasked with conserving marine biodiversity and moving fisheries toward sustainability [10]. Organisms inhabiting MPA networks can be viewed as a marine metapopulation, where subpopulations of relatively sedentary adults are connected by a widely dispersing larval stage. One of the purported ecosystem services provided by MPA networks is the ability of protected area populations to seed non-protected area populations through larval dispersal. Yet despite its importance, evidence for this larval reseeding remains elusive.

One reason for this lack of data is the great logistical challenges associated with tracking mm-scale larval fish subject to advection along open coastlines [11]. Therefore robust estimates of population connectivity among MPAs remain largely in the realm of simulation modeling (e.g. [12]–[15]). In southern California simulation models have shown connectivity patterns over shorter time scales (i.e. <30 days) are heterogeneous, but on longer time-scales connectivity patterns become homogenized [16]. Despite the stochastic nature of predictions from these simulation models, recent findings suggest that dispersal of some larval fishes may be cohesive rather than random (i.e. cohorts of larval fish may disperse and settle together) [17], [18]. An outstanding challenge to validating simulation-based estimates of connectivity are empirical data describing the swimming behavior of larval fish during dispersal. Due to the vertically stratified nature of currents in the marine environment, predictions of population connectivity vary widely depending on where individuals disperse within the water column. Developing this mechanistic understanding of connectivity and its role in population dynamics will require empirical evidence [3], [19]–[21].

Recent molecular studies have shed some light on the magnitude of self-recruitment and connectivity in insular settings; particularly in tropical systems. High self-recruitment was documented within populations of two coral reef fishes inhabiting a MPA at Kimbe Island, Papua New Guinea, and provided the first unequivocal documentation of fish dispersing among locations for a suite of proposed MPAs [22], [23]. Self-recruitment within this embayment was ∼40% and larvae dispersed up to 35 km over an 11-day pelagic larval period. In a follow-up study, [24] showed these approximate levels of self-recruitment remain consistent across years, despite variability in connectivity patterns between species and MPAs. Christie et al. [25] used parentage analysis to assess connectivity among surgeonfish inhabiting coral reefs in Hawai’i. This study documented low levels of self-recruitment, larval dispersal distances between 15 and 184 km, and larvae dispersed from MPAs to two non-MPA reefs [25]. While these studies present strong support of self-recruitment and connectivity in insular settings, evidence of these processes along continental coastlines with prevailing directional currents remains elusive.

Some studies attempting to quantify self-recruitment and connectivity along continental coastlines have met with some success. Two mussel species exhibit self-seeding within and connectivity among broadly defined regions along the open coastline of southern California [26]. Differing reproductive seasons (spring vs. fall) combine with reversal of oceanographic currents to yield different dispersal trajectories for these two co-occurring species [19]. In one of the few studies exploring dispersal patterns of a temperate reef fish, Di Franco et al [27] utilized otolith microchemistry to quantify natal origin and site fidelity around MPAs sited along the southeast coast of Italy. Individuals appeared to have originated from three distinct spawning regions spanning ∼200 km, but following settlement approximately 1/3 of individuals recruited into the adult population at their settlement site.

Building upon the foundation laid down by earlier researchers, we have applied otolith microchemistry to quantify self-recruitment and connectivity among a network of MPAs and non-MPA sites located along the open coastline of the northeast Pacific Ocean. *Hypsypops rubicundus*, a temperate damselfish, was selected as our model species; it is one of the most numerically abundant demersal species inhabiting nearshore rocky reefs within the study region [28]. *H. rubicundus* was selected as the most appropriate model species because: (1) it has life-history traits similar to numerous inshore rocky reef fishes, (2) it is an abundant species because it has been protected for over 30 years (i.e. avoiding the shifting baseline syndrome (see [29]) and (3) it has benthic nests amenable to the collection of embryonic fish necessary for microchemical analysis (see below). Using *H. rubicundus*, we capture the contrast between bi-weekly and annual connectivity patterns, and highlight the challenges and conservation ramifications of these for MPA design. More specifically, high-resolution sampling over a three-month spawning season enabled an exploration of how patterns of population connectivity change over three distinct time scales: biweekly, seasonally, and inter-annually.

This study was designed to answer two questions fundamental to the success of MPAs as management tools: (1) are San Diego coastal MPAs connected by larval dispersal? and (2) does self-recruitment occur along the open coastline of San Diego County? In addition, it enabled us to generate estimates of dispersal distance, dispersal directionality, comparison of source populations identified during subtidal surveys with predictions derived from otolith microchemistry, and conduct a *post hoc* comparison of population connectivity patterns estimated from microchemistry with depth-specific simulations of dispersal based on flow data from an Acoustic Doppler Current Profiler (ADCP). This enabled a test of the efficacy of logistically less intensive ADCP measurements as proxies for larval dispersal, and generated predictions of putative larval dispersal depths that can be tested in subsequent studies. By addressing these questions, and exploring the magnitude and variability of larval exchange among a reef network we provide empirical data critical for the validation of theoretical and simulation-based predictions of MPA connectivity, ultimately bettering our stewardship of marine resources.

## Methods

### Model Species

The natural history of *H. rubicundus* (Pomacentridae) in San Diego waters has been described in detail [30], [31]. Briefly, spawning females deposit eggs on male-guarded benthic nests between May and August. During the three-month spawning season clutches of embryos develop on benthic nests for two weeks; subsequently embryonic fish hatch and disperse for 18–22 days before settling to shallow rocky reefs [31]–[33].

### Identification of Putative Source Populations

**Figure 1:**
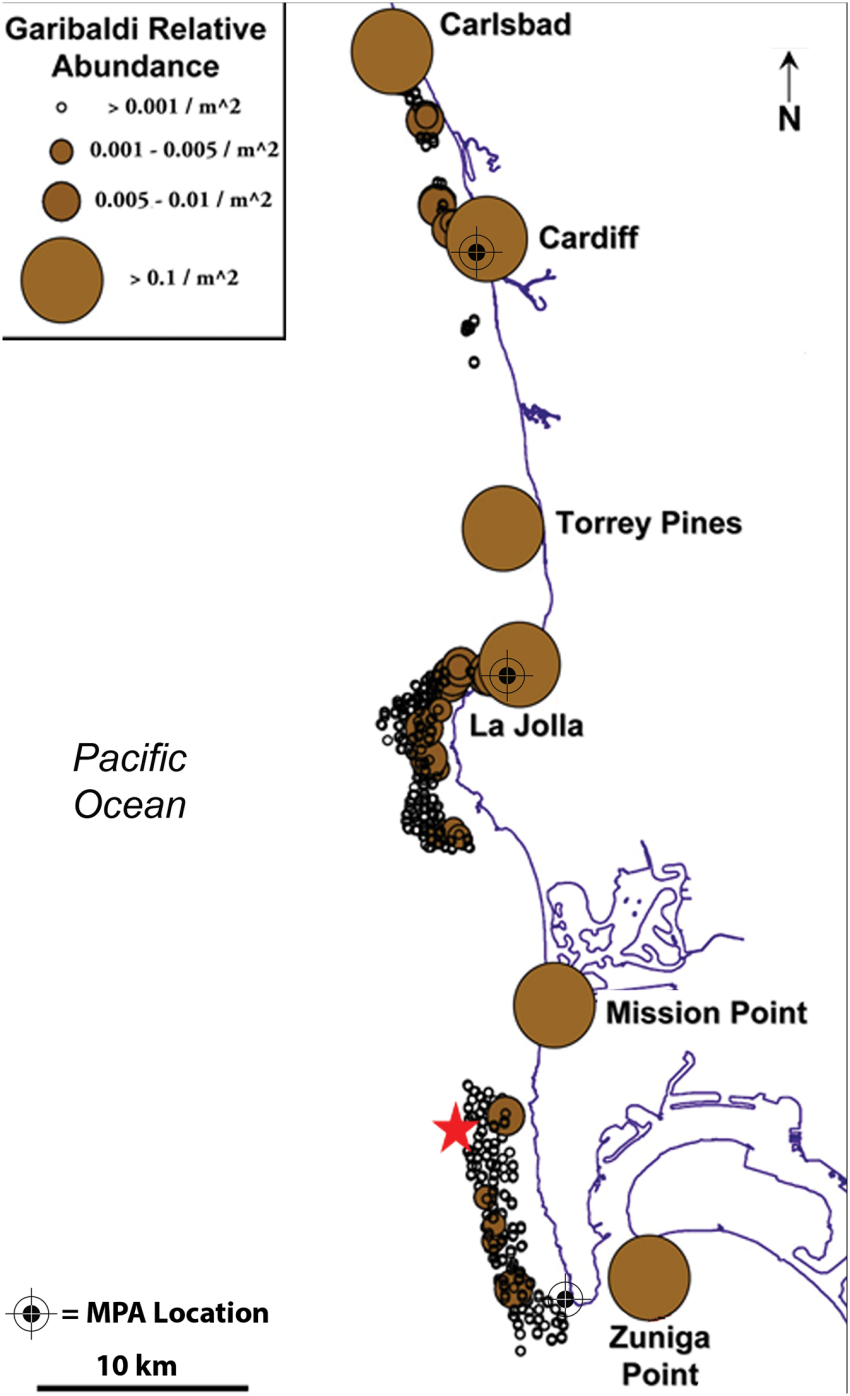
Map of study area with relative abundance of model species, *H. rubicundus* and location of MPAs. Names and locations of study sites are provided; approximate locations of MPAs are identified by black target symbols. Zuniga Point is adjacent to the Cabrillo State Marine Reserve. Location of ADCP (∼32.7°N, 117.3°W) is indicated with a red star. Regions appearing to be void of individuals are predominantly low-relief sandy bottom habitat and therefore do not support populations of *H. rubicundus*.

As part of the California Marine Life Protection Act there was an effort to create a network of MPAs spanning the southern California coastline [34]. In January 2012 after three years of study the new MPA network went into effect, providing some form of protection to approximately 15% (∼920 km^2^) of the south coast region. The geographic extent of our study included the entirety of south coast subregion 5, spanning from ∼Carlsbad, USA to the USA-Mexico border (Figure 1). More than 600 surveys were conducted using SCUBA between 2002 and 2008 to identify possible source populations of *H. rubicundus* inhabiting nearshore rocky reefs spanning the entire south coast study region. This region is comprised of patches of rocky reef separated by vast (i.e. kms to 10 s of kms) relatively flat sandy bottom habitat. Densities of *H. rubicundus* were highest where rocky substrate had vertical relief ≥1 m; vast sandy bottom regions separating rocky reefs were uninhabited (Figure 1). The core study region spanned ∼60 km; subtidal surveys conducted as part of this project indicate the nearest possible source populations outside of this region were >50 km to the north and >30 km to the south (Figure 1; please see [28] for a detailed description of subtidal survey methodology). Estimates of passive dispersal (see *Flow Based Predictions of Larval Dispersal* and associated text below) were used to quantify the uncertainty associated with the possibility a young-of-the-year settler sampled within the study region originated at an unsampled site beyond the study domain, and how this uncertainty could impact the interpretation of the connectivity results. Within the focal study region, six sites were identified as putative source populations; alone they support >90% of the *H. rubicundus* population within the study region and represent >99% of the putative population defined by microchemistry methods [28], [35]. Of these sites, two are within the boundaries of MPAs, Cardiff and La Jolla, and one, Zuniga Point, is adjacent to a third MPA, the Cabrillo State Marine Reserve (Figure 1).

### Empirically Quantifying Larval Dispersal and Population Connectivity

Otolith microchemistry was used to infer larval dispersal distances and generate population connectivity estimates. Otoliths (i.e. ear-stones) are aragonitic structures found in fishes. As otoliths grow, elements from the surrounding water mass become incorporated into the aragonite matrix, creating a chemical fingerprint that can be used to distinguish among geographic regions [36], [37].

Throughout the spawning and settlement seasons (i.e. May–November) all study sites were visited biweekly and surveyed for nests and/or young-of-the-year (YOY). If present, late-stage (i.e. just prior to hatching) embryonic fish and/or YOY were collected. In this study YOY refer to individuals recently settled to the reef; mean (±se) standard length of YOY was 24.3 mm (±0.8 mm) [28]. Samples were collected under California Department of Fish and Game scientific collecting permit #SC-009784. During subtidal collections at study sites all YOY observed were captured when present. Since sampling effort was similar across all sites, we assume the YOY collected and ensuing connectivity patterns represent the same origins and connectivity patterns as the larger unsampled portion of the population. Therefore we present our connectivity results as a proportion of all individuals dispersing within the study region. Data describing number of samples collected and analyzed at each location for 2008 and 2009 can be viewed in Table 1. Individuals were collected as still encapsulated embryonic fish affixed to benthic nests. However, embryonic individuals usually hatched and were actively swimming larval fish by the time a diver finished collections (in <2 hours) and returned to the surface. Otoliths from these hatched larval fish were extracted and analyzed (*n* = 1784), their microchemistry was analyzed, and algorithms were used to create a chemical reference map of the study region for each two-week period (i.e. a characterization of the study sites based upon their unique microchemical signatures; see below). Later in the spawning season (typically August), post-dispersal YOY appear on rocky reefs. These YOY were collected (*n* = 89), and similar to the larval fish, their otoliths were extracted. Overall settlement to reefs was episodic. Across all study sites the total number of settlers was much greater in 2008 than in 2009; despite similar sampling efforts between years it was possible to collect only 17 YOY in 2009 (i.e. all individuals that settled to study sites were collected and analyzed in 2008 and 2009). After processing, the microchemistry of the otolith core (i.e. the natal portion) was analyzed. The natal origin of YOY was inferred by comparing the otolith core chemical signature against the appropriate chemical reference map (i.e. the two-week period when the YOY was an embryonic fish developing on a benthic nest). These data were used to quantify connectivity among sites and to estimate larval dispersal distances and rates.

**Table 1:** Number of nests collected, otoliths analyzed, mean (±SD) and number of otoliths analyzed per nest, range of spawning season, and number of young-of-the-year (YOY) collected at each reef.

	Study Reef						
	Carlsbad	Cardiff	Torrey Pines	La Jolla	Mission Point	Zuniga Point	Total
NestsCollected 2008	10	12	32	18	11	12	95
NestsCollected 2009	23	21	26	17	21	26	136
Larval FishOtoliths Analyzed2008	80	114	456	212	123	116	1101
Larval FishOtoliths Analyzed2009	118	107	132	101	100	125	683
Otoliths Analyzedper Nest 2008	9.5±2.5	10.8±5.0	19.8±5.9	10.9±4.9	14.8±7.0	9.5±3.0	
Otoliths Analyzedper Nest 2009	6.9±4.9	7.1±5.2	10.2±6.0	7.2±6.0	7.7±6.4	6.6±6.9	
Range of 2008Spawning Season	July 24 -	July 10 -	June 03 -	June 03 -	July 15 -	July 22 -	
	21-Aug	21-Aug	02-Sep	29-Jul	12-Aug	19-Aug	
Range of 2009Spawning Season	June 11 -	June 11 -	May 29 -	May 29 -	June 12 -	June 12 -	
	26-Aug	11-Aug	26-Aug	29-Jul	12-Aug	28-Aug	
YOYCollected 2008	18	10	0	42	1	0	72
YOYCollected 2009	9	1	0	6	0	1	17

### Otolith Processing

Methods for otolith extractions and processing of larval and YOY fish otoliths were as follows. Individual sagittal otoliths were removed from randomly selected larval fish using fine-tipped tungsten probes; sagittal otoliths from YOY were removed with ceramic scalpels and manipulated with Teflon-coated forceps. Subsequently otoliths went through a series of cleaning, sonicating, and rinsing steps using 15% H_2_O_2_ buffered with 0.05 mol L^−1^ NaOH (to remove organic material) and MilliQ water (i.e. quartz-distilled water with resistivity >18.1 MΩ) in a Class 100 clean room. After the final cleaning step, larval fish otoliths were mounted on double–sided tape affixed to petrographic slides; YOY fish otoliths were mounted in cyanoacrylate on petrographic slides. After processing, YOY and larval fish otoliths were stored within sealed petri dishes and placed under a Class 100 laminar flow hood housed within a Class 100 clean room until analysis.

Right sagittal otoliths of YOY were used to estimate the period within the spawning season when individuals were developing on benthic nests. After polishing otoliths, light transmission microscopy was used to count daily growth rings. Counts were made outward from the edge of the natal region to an outer edge of the otolith. However the core region rings were frequently difficult to count due to poor light transmission. Therefore YOY were placed conservatively within 14-day nesting bins reflecting their putative two-week benthic embryonic period. The natal chemical signature of the left sagittal otolith removed from the same fish was compared with the chemical reference map from the corresponding period of the spawning season to infer natal origin (see [35] for detailed discussion of methodology).

### Laser Ablation – Inductively Coupled Plasma Mass Spectrometry

All microchemical analyses were conducted using a New Wave UP 213 nm laser ablation unit coupled to a Thermoquest Finnegan Element 2 Inductively Coupled Plasma Mass Spectrometer (LA-ICPMS) at the UC Santa Barbara MSI Analytical Lab.

Seven isotopes were selected for analysis: ^24^Mg, ^48^Ca, ^55^Mn, ^87^Sr, ^138^Ba, ^208^Pb, and ^238^U. However there is a minute probability that Pb was a source of contamination, therefore all results presented here exclude Pb (please see Discussion below). Individual isotopes were included in subsequent analyses if their relative concentration was greater than 3X the standard deviation of blanks run during each sequence. For 2008 samples, laser intensity was set at 50% with a 40-µm spot size and a 4 second dwell time. With 2009 samples, signal stability was highest when laser intensity was reduced to 30%, and dwell time was increased to 5 seconds. These laser parameters were selected to maximize signal intensity and stability, as determined during methods development. The laser spot size was selected based on measurements of larval fish otoliths. Larval fish otoliths ranged in diameter from 8.5–46.4 microns (mean = 36.5 (±2.3) microns; Cook, unpub. data). Due to this relatively small size, it was necessary to ablate the entire larval fish otolith and the entire natal core region of YOY otoliths to obtain signal stability and counts greater than detection limits. Estimates of instrument external precision and detection limits derived from consistency standards for 2008 and 2009 are provided in Table 2.

**Table 2:** Estimates of external precision (% relative standard deviation, RSD) for LA-ICPMS analyses and associated limits of detection for elements of interest.

2008% RSD and Detection Limits				
Element	OTO	NIST 612	MACS 3	Detection Limit
**Mg**	1.03%	9.50%	7.68%	0.04 mmol/mol Ca
**Mn**	1.63%	8.87%	6.02%	0.03 mmol/mol Ca
**Sr**	2.18%	8.23%	4.74%	0.04 mmol/mol Ca
**Ba**	3.22%	10.01%	8.93%	0.31 µmol/mol Ca
**Pb**	4.41%	10.07%	8.17%	0.25 µmol/mol Ca
**U**	5.27%	8.68%	13.43%	0.01 µmol/mol Ca
**2009% RSD and Detection Limits**				
**Element**	**OTO**	**NIST 612**	**MACS 3**	**Detection Limit**
**Mg**	1.90%	3.84%	3.79%	0.06 mmol/mol Ca
**Mn**	1.95%	2.75%	6.83%	0.02 mmol/mol Ca
**Sr**	1.77%	3.18%	7.29%	0.13 mmol/mol Ca
**Ba**	1.77%	3.05%	14.43%	0.49 µmol/mol Ca
**Pb**	2.14%	3.27%	12.49%	0.36 µmol/mol Ca
**U**	3.67%	2.48%	17.89%	0.04 µmol/mol Ca

RSDs are presented for a dissolved CaCO3 reference material (OTO), and two solid reference materials: NIST 612 and USGS MACS3. Units for “concentrations” of elements are given as ratios to ^48^Ca.

### Otolith Microchemistry

Microchemistry data were assessed for normality and homogeneity of variances, and where warranted, outliers (i.e. samples falling beyond 95% confidence intervals [38]) were examined further and when deemed necessary removed from analyses. Outliers were removed when 1) lower analytical precision values (i.e. high RSD) were reported following laser ablation of a sample, 2) possible spurious sample data (e.g. a note indicated sample was relatively small (>10 µm in diameter), or 3) laser ablation proceeded in an abnormal manner (e.g. at times otoliths appeared to be knocked out of field of view when laser ablation commenced). Microchemistry data were not multivariate normal despite multiple transformations. Multivariate outliers were identified using jack-knifed Mahalanobis distances. Outliers (as identified above) were 20 of 1101 reference larval fish otoliths from 2008 and 7 of 683 larval fish otoliths from 2009; in total 1.5% of all larval fish otoliths were removed as outliers. All 89 YOY collected across both years were used in analyses.

Previous research has shown embryos collected <4 km apart are indistinguishable; for this study microchemistry data are assumed to represent all individuals residing within ∼4 km of the primary study sites [28]. Temporally, data were binned fortnightly; this is the period of time in which the embryonic fish acquire their natal signatures [35]. To assess chemical differences among reefs, linear discriminant function analysis (DFA) was used. Linear DFA is relatively robust to violations of multivariate normality and analysis of the discriminant functions supported the assumption of homogeneity of the variance-covariance matrices [39]. All DFAs were run in a stepwise manner; only elements with an F-to-remove statistic greater than 2.5 (p<0.05) were included. For comparative purposes and to assign significance levels to the DFA classification success, data were compared against the classification success generated from 1000 randomized data sets (i.e. a null expectation of classification success); all DFAs and randomization procedures were run in Matlab Version 8.1, with code modified from [40]. Maximum likelihood estimation was used to generate connectivity estimates of error and to classify YOY to natal region using HISEA [41], [42] following the methods of [43]–[45]. The direct maximum likelihood estimator was used in all cases. In these analyses the larval fish otoliths collected in each biweekly spawning bin were used as a baseline and were resampled 2000 times to generate estimates of classification error. The YOY were considered a stock mixture of unknown origin, and HISEA was used to estimate the source populations contributing to the YOY mixture. However, HISEA does not provide origins of individual fish (only the proportion of a mixed stock belonging to the possible source populations [45], [46]). Therefore we present these findings as an additional line of evidence along with the DFA classification results of individual YOY.

### Population-Normalized Estimates of Connectivity

To explore patterns of population connectivity relative to adult population size we calculated mean adult density at each of the six primary study sites from SCUBA transect surveys (n = 647). From these transect data we calculated mean (±se) adult and maximum YOY density at each study site; maximum YOY density was the calculated as the greatest density following a settlement pulse. The expected number of YOY originating from each site was estimated as a function of the adult population size at each study site. A chi-square goodness of fit test was used to test whether the probability of the observed number of YOY originating from each source population was significantly different from that expected by chance (i.e. assuming all sites contributed YOY in proportion to their adult population size).

### Flow-Based Predictions of Larval Dispersal

Acoustic Doppler Current Profiler (ADCP) data generated as part of an independent study on southern California kelp forest ecosystems was used to develop hypotheses about vertical location of dispersing larval fish. Currents were measured between May 2008 and September 2009. An RDI 660 kHz ADCP was deployed on the ocean bottom at a depth of ∼32 m between Zuniga Point and Mission Point (Figure 1). The ADCP sampled data in 1 minute ensembles over 2-m depth bins between May 7, 2008 and May 31, 2008, and in 5 minute ensembles thereafter. For this study, current data were lowpass filtered to remove the tides (<2 days) using a Butterworth filter. Currents were then plotted in depth/time space with color indicating direction and opacity (i.e. intensity) indicating magnitude. Mean current magnitudes were also plotted by bin for each summer after decimating the one-minute data from the first deployment to 5 minutes using a Chebyshev Type I filter. Data were analyzed using the Matlab digital signal processing toolbox. No inshore current data were available from other areas off San Diego for the study periods. However, previous concurrent data collected at La Jolla and the ADCP location indicated subtidal period currents are typically significantly correlated throughout the water column between the two areas (Parnell, unpub. data). Therefore we assumed the current regime at the ADCP location was similar across the study region.

Potential passive larval dispersal was estimated for each two-week nesting bin (i.e. the two week period when larval fish were developing otolith trace elemental signatures as embryos on benthic nests) and compared to dispersal distances estimated from otolith microchemistry. Larval dispersal was simulated utilizing a continuous release of 1×10^6^ virtual larvae at each of 4 depth bins (4 m, 8 m, 12 m, and 16 m), assuming a Gaussian release kernel centered over the mid-point of each two-week benthic nesting period (e.g. for the June 01–June 15 nesting bin, the peak in the virtual release was centered around June 08, and larvae released on this date would disperse until June 28). The release kernel simulated the empirically estimated pelagic larval duration, and we assumed no dispersion or larval mortality. Larvae were subjected to currents (progressively additive vectors) from the ADCP data (as described above) for 20 days utilizing a reflecting shoreline. At day 20 the density of virtual larvae at a given distance for each depth bin was calculated. To quantify the uncertainty associated with the microchemistry estimates of natal origin, ADCP estimates of passive dispersal within the study region were extrapolated beyond the study domain to place bounds on how far individuals from the nearest source populations outside of the study domain may have dispersed during a portion of the study, and the probability that they infiltrated the study domain resulting in misclassification of natal origin. While these ADCP estimates cannot be substantiated, they provide a manner of estimating uncertainty of natal origin not captured by existing classification methods.

## Results

### Larval Dispersal and Natal Origins

The microchemistry of 1757 larval fish otoliths was used to create biweekly chemical reference maps for the six primary study sites across the 2008 and 2009 spawning seasons (∼May–August; Table 3, Table 4). The natal origins of 89 post-dispersal YOY were determined by comparing their otolith core microchemistry (i.e. the natal portion) with the chemical reference maps generated from the microchemistry of larval fish otoliths collected from the 6 study sites. Mean biweekly classification success of larval fish-derived chemical reference maps was 71%±4% (range = 58%–83%) in 2008 and 68%±5% (range = 60%–89%) in 2009. Across both years of this study mean accuracy of biweekly classification success of larval fish chemical reference maps as assessed using HISEA maximum likelihood estimation was ±5% (range ±3% to ±7%; Table 3). Mean classification success of post-settlement YOY was 96% (±4%) in 2008 and 97% (±3%) in 2009. All classification accuracies are significantly greater than jackknife re-classification success expected from randomized data sets (mean = 17–24%, p<0.001; following [40]).

**Table 3:** Biweekly classification success of larval fish otoliths and young-of-the-year (YOY) fish otoliths.

		Larval Fish				Young-of-the-Year Fish			
Year	Nesting Bin	LarvalOtoliths(n)	Elementsused in DFAClassification	DFAClassificationSuccess	HISEASimulationMLE SD	YOYClassified(n)	HISEA MLEPredicted Origin(YOY proportionof stockmixture)	NumericalEquivalentof YOY	HISEA MLESD (2000 BootstrapRuns)
2008	May 16–31	0	na	na	na	0	-	-	-
2008	June 1–15	67	Mg, Sr, Ba, U	79%	0.03	7	0.71 LJ, 0.29 TP	5 LJ, 2 TP	0.02
2008	June 16–30	99	Mg, Sr, Ba, U	83%	0.03	14	1.0 LJ	14 LJ	0.03
2008	July 1–15	134	Mg, Sr, Ba, U	72%	0.05	32	0.03 MP, 0.97 LJ	1 MP, 31 LJ	0.03
2008	July 16–31	276	Mg, Sr, Ba, U	66%	0.05	11	1.0 LJ	11 LJ	0.001
2008	Aug 1–15	202	Mg, Sr, Ba, U	69%	0.04	8	1.0 MP	8 MP	0.04
2008	Aug 16–31	303	Mg, Sr, Ba, U	58%	0.05	0	-	-	-
	2008 Total	1081		71.2%	0.04	72			0.02
2009	May 16–31	54	Mg, Sr, Ba, U	89%	0.05	2	1.0 LJ	2 LJ	0.001
2009	June 1–15	175	Mg, Sr, Ba, U	65%	0.06	1	-	-	-
2009	June 16–30	108	Mg, Sr, Ba, U	60%	0.05	0	-	-	-
2009	July 1–15	104	Mg, Sr, Ba, U	66%	0.03	7	1.0 LJ	7 LJ	0.001
2009	July 16–31	110	Mg, Sr, Ba, U	62%	0.06	5	0.79 MP, 0.21 ZP	4 MP, 1 ZP	0.07
2009	Aug 1–15	66	Mg, Sr, Ba, U	76%	0.03	2	1.0 CF	2 CF	0.08
2009	Aug 16–31	59	Mg, Sr, Ba, U	61%	0.07	0	-	-	-
	2009 Total	676		68.4%	0.05	17			0.04
2008–2009 Total		1757		69.7%	0.05	89			0.03

Estimates of variation and predicted stock mixture (i.e. proportional natal origin of stock) were generated using HISEA maximum likelihood estimation (MLE; Millar 1987, 1990). During June 1–15 2009 no MLE prediction of stock mixture was possible due to insufficient sample size (i.e. n = 1). Sites of origin are defined as follows: CF = Cardiff, LJ = La Jolla, TP = Torrey Pines, MP = Mission Point, and ZP = Zuniga Point.

**Table 4:** Connectivity matrices for 2008, 2009, and 2008–2009.

	2008 Population Connectivity Matrix					
	Carlsbad	Cardiff	Torrey Pines	La Jolla	Mission Point	Zuniga Point
Carlsbad	0	0	0	0	0	0
Cardiff	0	0	0	0	0	0
Torrey Pines	1	0	0	3	0	0
La Jolla	15	8	0	36	0	0
Mission Point	2	2	0	4	1	0
Zuniga Point	0	0	0	0	0	0
	**2009 Population Connectivity Matrix**					
	**Carlsbad**	**Cardiff**	**Torrey Pines**	**La Jolla**	**Mission Point**	**Zuniga Point**
Carlsbad	0	0	0	0	0	0
Cardiff	0	0	0	2	0	0
Torrey Pines	0	0	0	0	0	0
La Jolla	5	0	0	3	0	1
Mission Point	3	1	0	1	0	0
Zuniga Point	1	0	0	0	0	0

Values represent numbers of YOY fish. Rows are predicted natal reefs; columns are reefs where post-dispersal YOY were captured.

**Figure 2:**
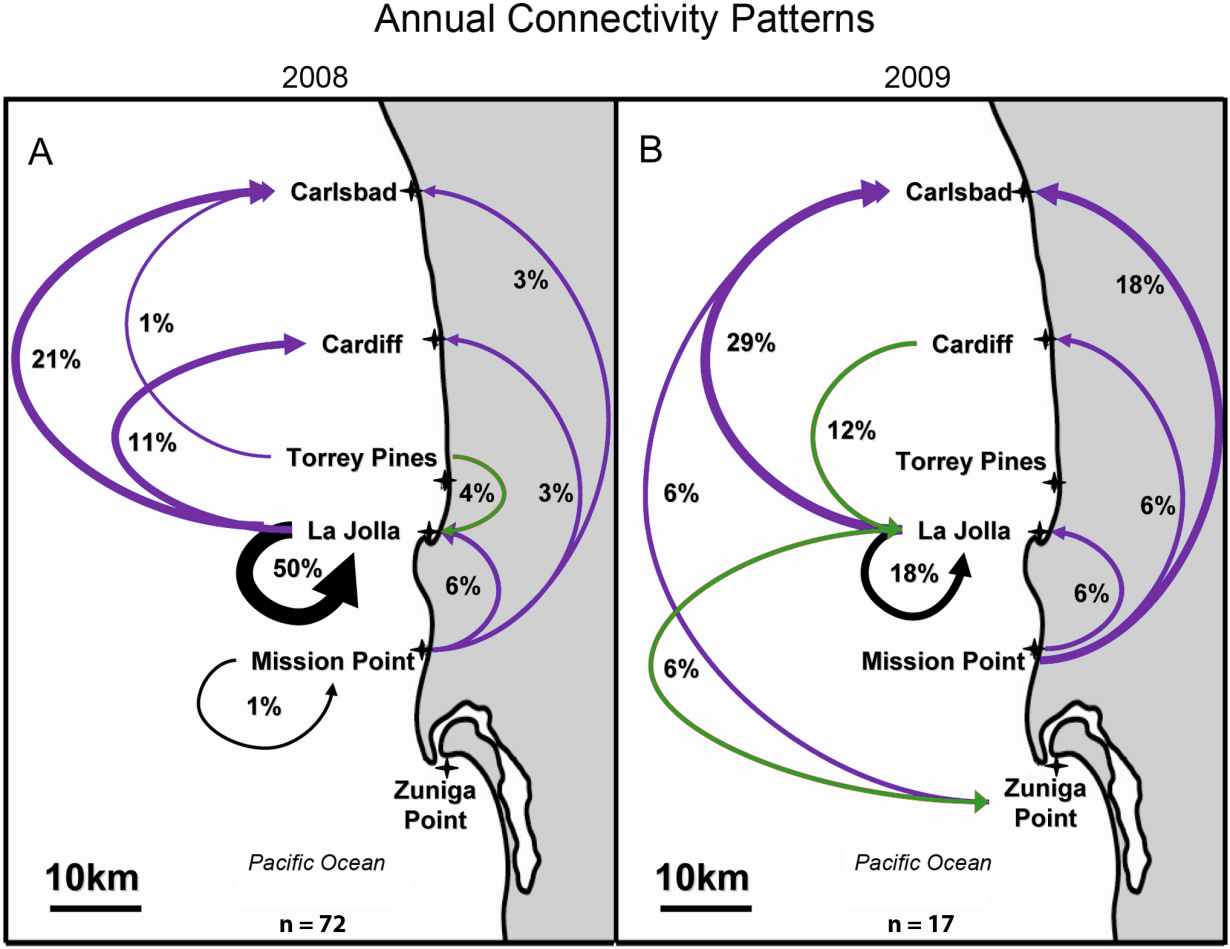
Aggregate annual connectivity patterns in 2008 (Fig. 2A) and 2009 (Fig. 2B). Grey region is land and white region is ocean. Arrows indicate predicted larval dispersal trajectories from natal origin to capture location of post-settlement YOY. Purple arrows indicate northward dispersal, green arrows indicate southward dispersal, and black arrows indicate self-recruitment. Thickness of arrows is proportional to the number of individuals dispersing among reefs. Annual sample sizes of YOY (*n*) are provided at the bottom of each sub-panel (e.g. the natal origins of 72 post-settlement YOY were determined in 2008). Percentages in each sub-panel indicate the proportion of YOY dispersing within the entire study system. For example, in 2008, 36 of 72 (i.e. 50%) of YOY collected at La Jolla appear to have self-recruited. Crosses indicate location of primary study sites.

In 2008, YOY (*n* = 72) originated from one of three natal reefs: Torrey Pines, La Jolla, or Mission Point (Table 3, Table 4). Of these locations, the sole no-take MPA (i.e. La Jolla) was the largest source population; 82% (i.e. 59 of 72) of all captured YOY were predicted to have come from La Jolla. Mission Point and Torrey Pines produced 13% (9 of 72) and 5% (4 of 72) of post-dispersal recruits, respectively (Figure 2A, Table 3). Net dispersal distance ranged between 0 km and 46 km over a 20-day pelagic larval period. When dispersal distances from all YOY were combined (individuals self-recruiting to a reef were assigned a net dispersal distance of 0 km), mean (±se) dispersal distance in 2008 was 12.3 km (±1.8 km). The mean dispersal distances for larvae originating from each of the three source populations, Torrey Pines, La Jolla, or Mission Point was 10.5 (±7.1) km, 10.7 (±1.8) km, and 23.6 (±4.7) km, respectively. Larval fish originating at Mission Point dispersed significantly farther than those originating in La Jolla (One-way ANOVA F_2,69_ = 3.23, p<0.05; Tukey HSD p<0.05). On biweekly time-scales, mean dispersal distances differed significantly over the course of the 2008 spawning season; mean dispersal distance for larvae that were developing embryos during August 1–15 2008 (24.8±4.9 km) was greater than those developing during July 16–31 2008 (5.8±4.2 km) (One-way ANOVA F_4,67_ = 2.7 p = 0.04, Tukey HSD p<0.05). Excluding self-recruiting individuals, and assuming an 18–22 day pelagic larval period, this suggests mean larval transport rates in 2008 ranged from 0.26 cm s^−1^ to 2.96 cm s^−1^. When directionality of larval dispersal was assessed, 44% of larval fish dispersed in a northerly direction (on average 27.2 km±1.53), 51% self-recruited, while the reminder (5%) dispersed in a southerly direction (on average 5.0±0 km).

In 2009, four of the six reefs were natal origins of 17 post-settlement stage fish: Cardiff, La Jolla, Mission Point and Zuniga Point (Table 3, Table 4). La Jolla was again the predominant source of post-dispersal fish within the study region, producing 53% (n = 9) of post-settlement YOY. Mission Point, Cardiff, and Zuniga Point produced 29% (n = 5), 12% (n = 2), and 6% (n = 1) of YOY, respectively (Figure 2B, Table 3). In 2009 individual fish dispersed between 0 km and 59 km; mean larval dispersal distances from Cardiff, La Jolla, Mission Point, and Zuniga Point were 19.0 (±10.3) km, 20.8 (±4.9) km, 36.8 (±6.5) km, and 59.0 (±14.6) km, respectively. Making the same assumption regarding pelagic larval duration, and excluding individuals that self-recruited, empirical data suggest mean net larval transport rates in 2009 ranged between 0.74 cms^−1^ and 3.79 cms^−1^. When directionality of dispersal was assessed for 2009, 65% of YOY dispersed in a northerly direction (36.6±3.6 km), 18% self-recruited, and 17% dispersed in a southerly direction (21.7±2.7 km).

**Figure 3:**
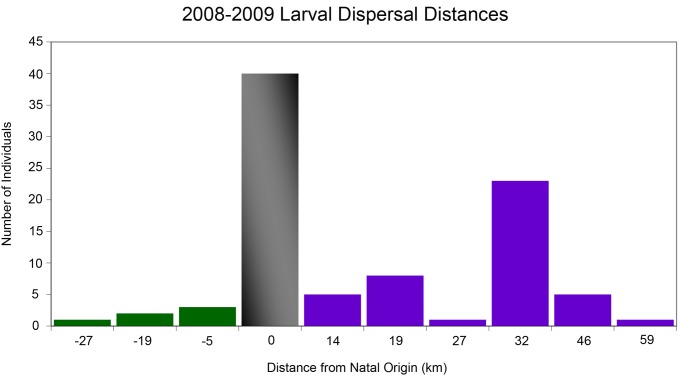
Mean larval dispersal distances (km) of all (n = 89) YOY fish collected in 2008 and 2009. Green histograms and negative values indicate southward dispersal. Purple histograms and positive numbers indicate northward dispersal, and the grey histogram depicts the number of YOY that self-recruited (i.e. that were spawned at and settled to the same reef).

When comparing larval dispersal distances between years, *H. rubicundus* larvae dispersed significantly longer distances in 2009 (27.5 km) than in 2008 (12.3 km; One way ANOVA F_1,87_ = 13.9, p<0.001). When locations were pooled across years, larval fish from Zuniga Point dispersed significantly greater distances (59.0 km) than those originating from La Jolla or Torrey Pines (12.4 km or 10.5 km, respectively; Tukey HSD p<0.05). Including individuals that self-recruited, mean larval dispersal distance across both years of the study was 15.2 km (±1.7; Figure 3).

### Population-Normalized Estimates of Connectivity

Based on adult fish densities obtained from transect surveys, and assuming source locations contribute YOY in proportion to local adult population size, data suggest individual sites would contribute between 6% (∼6 individuals; Cardiff) and ∼25% (∼22 individuals; Carlsbad) of YOY within the six-site metapopulation (Table 5). However, observed numbers of YOY from individual sites ranged from 0% (Carlsbad) to 76% (La Jolla); the observed number of YOY settling to each site was significantly higher (or lower) than that expected by chance (χ^2^_(5,89)_ = 273.9, p<0.0001; Table 5), suggesting that within the study region the proportion of YOY originating from each site could not be predicted by knowledge of adult source population size.

**Table 5:** Adult and YOY density at six primary study sites and within the three broad geographic regions surveyed: North County, La Jolla, and Point Loma.

Site	Number ofDives/Surveys	Adult Densityind./m^2^ (±1 se)	AdultDensityTukey HSD(p<0.001)^a^	2008 Max.YOYDensity(ind./m^2^)	2009 Max.YOYDensity(ind./m^2^)	Observed #of YOYoriginatingfrom site	Expected #of YOYoriginatingfrom sitebased onadult density
Carlsbad	15^b^	0.346 (0.007)	A	0.05	0.02	0	22
Cardiff	16^b^	0.089 (0.007)	E	0.02	0.008	2	6
Torrey Pines	21^b^	0.335 (0.006)	A	na	0.008	4	21
La Jolla	23^b^	0.212 (0.006)	C	0.21	0.02	68	13
Mission Point	16^b^	0.155 (0.007)	D	0.008	na	14	10
Zuniga Point	14^b^	0.276 (0.007)	B	na	0.008	1	17
North County Region	70^c^	0.013 (0.003)	F	na	na	-	
La Jolla Region	264^c^	0.004 (0.002)	F	na	na	-	
Point Loma Region	208^c^	0.0008 (0.002)	F	na	na	-	

Maximum YOY density was calculated using the maximum number of YOY recruits observed on a 120 m∧2 belt transect after a settlement pulse. na indicates ‘not applicable’ (i.e. no YOY were present).

a Sites not connected by similar letters have significantly different densities (One-way ANOVA F_8,638_ = 936.9, p<0.00001; Tukey HSD p<0.001).

b Two replicate belt transect surveys 120 m∧2 (30 m×4 m) were conducted per dive.

c Surveys in broad geographic regions (North County, La Jolla, Point Loma) were conducted between March and June prior to settlement of YOY and consisted of 2–4 belt transect surveys per site.

### Connectivity and Self-Recruitment in MPAs

#### MPA Connectivity

In 2008 two of the three MPAs, Cardiff and La Jolla, were connected to one another by larval dispersal; Zuniga Point was not a source or destination of any YOY (Figure 2A). Assuming the YOY captured reflect the broader population, we estimate 80% of the YOY at Cardiff were supplied by La Jolla, while the remaining 20% were from Mission Point. In 2009, however, this connectivity pattern was reversed; 33% of the YOY settling in La Jolla originated from Cardiff, while none of the YOY settling at Cardiff were supplied by La Jolla (Figure 2). When MPA connectivity patterns were summed across 2008 and 2009, ∼9% of the YOY (*n* = 8) dispersed northward from La Jolla to Cardiff, 2% (*n* = 2) dispersed southward from Cardiff to La Jolla, and 1% (*n* = 1) dispersed southward from La Jolla to Zuniga Point. Excluding individuals that self-recruited, 36% of YOY dispersed between a non-MPA and a MPA reef or dispersed between two non-MPAs (8%). Including individuals that self-recruited, 56% (50 of 89) of all YOY settling within the study region contributed to MPA connectivity (i.e. 44% through self-recruitment to an MPA and 12% dispersing between two MPAs).

**Figure 4:**
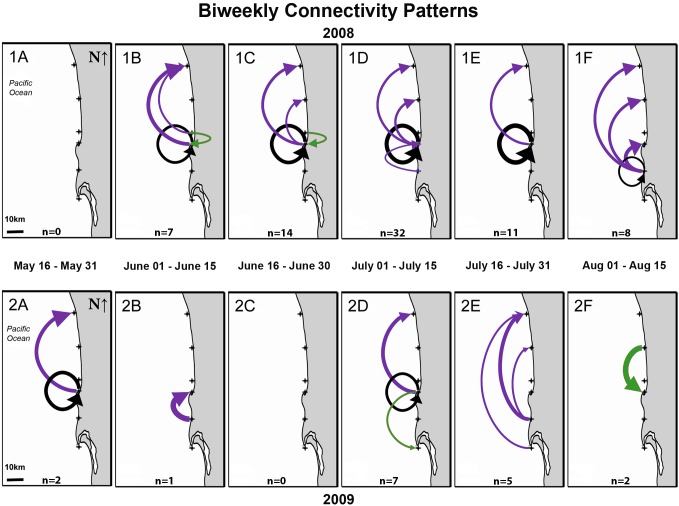
Bi-weekly connectivity patterns in 2008 (Fig. 4-1A-1F) and 2009 (Fig. 4-2A-2F). Dates indicate the two-week bin in which an embryonic fish was developing on a benthic nest (i.e. acquiring its natal elemental fingerprint). Grey region is land and white region is ocean. Arrows indicate predicted larval dispersal trajectories from natal origin to capture location of post-settlement YOY. Purple arrows indicate northward dispersal, green arrows indicate southward dispersal, and black arrows indicate self-recruitment. Thickness of arrows is proportional to the number of individuals dispersing among reefs. Numbers of YOY (*n*) dispersing among reefs are provided at the bottom of each sub-panel. For example, in Figure 4-1B (June 01, 2008–June 15, 2008) the connectivity patterns of 7 post-settlement YOY were recreated. Crosses indicate location of primary study sites.

Zuniga Point did not exchange larvae with any of the study reefs in 2008, but it did act as a source and destination for YOY in 2009. This location supplied ∼11% of the YOY to Carlsbad in 2009, a site almost 60 km to the north, and it received a single southward dispersing YOY from one of the other MPAs (La Jolla), a distance of almost 30 km. Viewed in isolated biweekly bins, it could appear Zuniga Point was not connected with other study sites, however protracted high-resolution sampling across the entire 2009 spawning season showed Zuniga Point acted intermittently as both the origin and destination of settlers within the greater metapopulation (Figure 4-2D, 2E).

#### MPA Self-recruitment

Over the course of the 2008 and 2009 spawning and settlement seasons, self-recruitment occurred at La Jolla and Mission Point; La Jolla was the only MPA within the study region to exhibit self-recruitment. In 2008, 84% (36 of 43) of the YOY collected in La Jolla appear to be self-recruits: these larvae represented 50% of all larval fish dispersing within the study region in 2008. In 2009, La Jolla was again the only location where self-recruitment occurred; self-recruitment supplied 50% of the YOY to La Jolla, but this value represented only 18% of all YOY collected within the study region (vs. 50% in 2008). Over shorter time scales La Jolla had relatively persistent self-recruitment over eight weeks of the 12-week spawning season in 2008 (Figure 4-1B-1E). In 2009 self-recruitment was more intermittent, occurring in late May, and again in early July (Figure 4-2A, 2D). Over both years of the study self-recruitment in MPAs accounted for 44% (39 of 89) of all YOY settling to rocky reefs; only one (of 89) YOY self-recruited to a non-MPA reef (i.e. Mission Point; Figure 2A, Figure 4–1F).

### Comparison of Otolith Microchemistry and Flow-Based Estimates of Connectivity

#### Nearshore Flow Patterns

**Figure 5:**
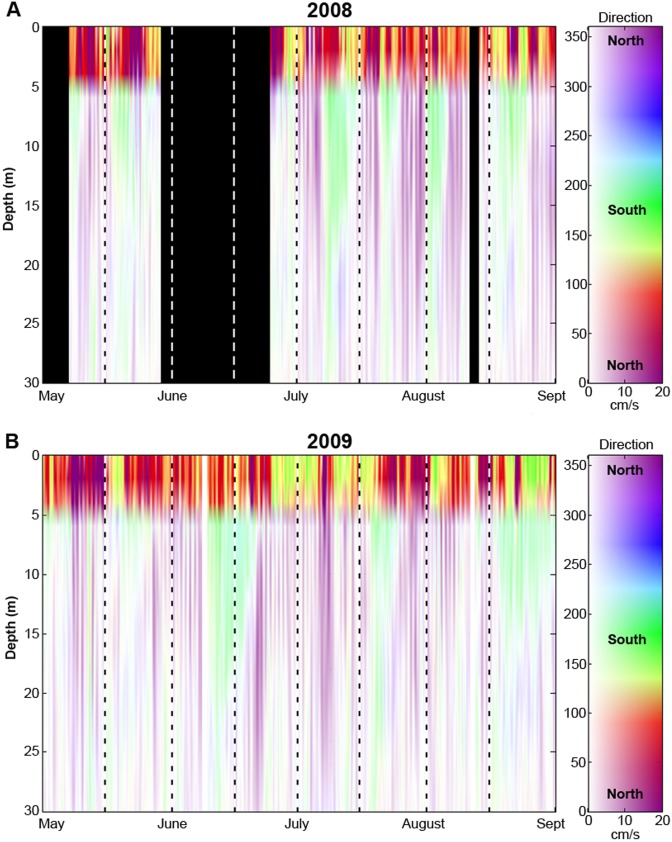
Acoustic Doppler Current Profiler (ADCP) data from 2008 (Fig. 5A) and 2009 (Fig. 5B). Purple colors indicate northerly flowing currents, green colors indicate southerly flowing currents, and red colors indicate onshore flow. Intensity of color represents magnitude of flow. Black regions represent periods of time when ADCPs were not collecting data.

Analyses of ADCP data show subtidal frequency currents deeper than ∼4 m were primarily alongshore (flowing approximately north-south with mean velocities of ∼2 cm sec^−1^) and occasionally exhibited vertical shear (Figure 5). These currents were dominated by unidirectional northward (purple in Figure 5) or southward (green in Figure 5) flow over periods of a few to ∼14 days. Near-surface currents were strongly affected by winds; mean current magnitudes in the top 4 m were an order of magnitude greater than subsurface currents and were directed mainly shoreward (red in Figure 5) and southward – the predominant direction of the afternoon sea breeze during summer.

**Figure 6:**
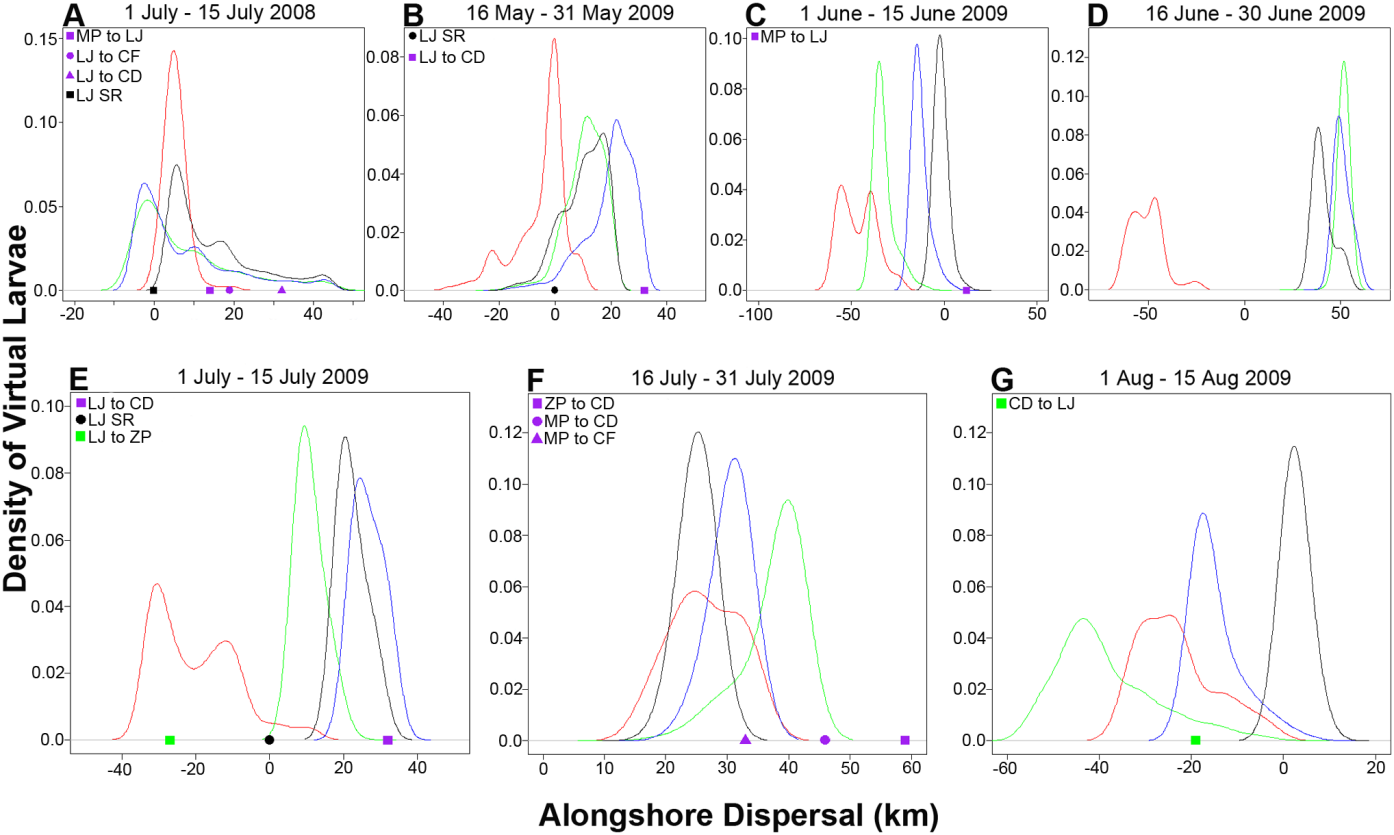
Progressive vector simulations of passive 20-day pelagic larval periods based on ADCP data. Simulations were conducted by continuously releasing (*n* = 1×10^6^) “virtual larvae” centered over the mid-point of each 14 day nesting bin: (Fig. 6A) July 01–July 15 2008, (Fig. 6B) May 16–May 31 2009, (Fig. 6C) June 01–June 15 2009, (Fig. 6D) June 16–June 30 2009, (Fig. 6E) July 01–July 15 2009, (Fig. 6F) July 16–July 31 2009, and (Fig. 6G) August 01–15 2009. Colored curves indicate probability density of alongshore virtual larval transport within 4 distinct depth bins over a 20 day pelagic larval period (red = 4–8 m, green = 8–12 m, blue = 12–16 m, black = 16–20 m). Positive values indicate northward transport, magnitude is in km. Symbols on the abscissa indicate larval dispersal estimates derived from otolith microchemistry. Colors of symbols represent directionality of dispersal: purple = northward dispersal, green = southward dispersal, black = self-recruitment. Study sites are defined as follows: CD = Carlsbad, CF = Cardiff, LJ = La Jolla, MP = Mission Point, ZP = Zuniga Point and SR = Self-Recruits. Please note different scales on axes.

Comparisons of ADCP-based and microchemistry based dispersal magnitude and directionality were possible for seven of the twelve two-week nesting bins spanning the 2008 and 2009 *H. rubicundus* spawning seasons: the July 1–July 15, 2008 nesting bin (i.e. the July 08–July 28 pelagic larval period) and all six of the 2009 2-week nesting bins (Figure 6). Microchemically-derived estimates of larval fish dispersal distances are an order of magnitude below the mean current velocities measured in surface waters (>20 cms^−1^) but fall within the range of velocities measured at water depths below 4 m (∼2 cms^−1^).

When otolith-derived estimates of connectivity are compared with ADCP data, there is a general concordance in directionality. For example, for individuals developing on benthic nests between July 01 and July 15 2009 (Figure 4-2D), microchemical data suggest individuals dispersed northward 32 km (*n* = 4), southward 27 km (*n* = 2), or self-recruited (*n* = 1). Depth-specific ADCP data show all three of these dispersal trajectories and magnitudes are feasible (Figure 6E); dominant southward flow was documented at 4 m water depth, self-recruitment was possible if the individual remained between 4 m and 8 m water depth, while northward flowing currents were found between 12 m and 16 m water depth. For embryonic fish developing on benthic nests between July 16–31, 2009 (Figure 4-2E), all individuals dispersed in a northerly direction and this two week nesting bin is followed by unilateral southerly dispersal from August 1–15 (Figure 4–2F). When ADCP data are viewed over these time periods a similar pattern is observed; currents flow in a northerly direction for a period of ∼3 weeks, followed by a reversal in current direction occurring at the approximate time the otolith-derived estimates suggest a change in dispersal direction (Figures 5B, 6F, 6G).

Within the seven two-week nesting bins where both microchemistry and ADCP data were available, there were 14 possible comparisons of dispersal magnitude and directionality (Figure 6). In these 14 pair-wise comparisons, the magnitude (distance) and directionality of dispersal predicted by the microchemistry data could be explained by ADCP data 93% of the time; there was one occasion when microchemistry predicted a dispersal distance greater than that predicted by ADCP data (Figure 6F, Zuniga Point to Carlsbad). This suggests that, with caution, ADCP data can generate hypotheses about larval dispersal depth, predict larval dispersal directionality, and when evaluated below wind-forced surface waters (i.e. >4 m water depth), resolve approximate larval dispersal distances for *H. rubicundus*.

## Discussion

### Uncertainty in Predicting Natal Origins

There are two primary sources of uncertainty when predicting natal origins. First, there is uncertainty associated with the classification method itself. As was mentioned in the text, the inherent variability within the larval fish otolith microchemistry dataset resulted in mean DFA classification success of 71.2% in 2008 and 68.4% in 2009 (see Larval Dispersal and Natal Origins above). When these probabilities are combined in a multiplicative fashion with the classification success of post-settlement YOY (96%±4% in 2008, 97%±3% in 2009, and 97%±4% across both years of this study), we can adjust the overall uncertainty of classification success (i.e. uncertainty = 1 - classification success) to 31% (2008) and 34% (2009). In both years classification uncertainty is significantly lower than the uncertainty of a jack-knifed randomized dataset (2008 = 76%, 2009 = 83%), and classification success is equivalent to many recent microchemistry studies (e.g. [27], [43]–[45], [47]). The HISEA MLE provides an additional supporting line of evidence in this regard; the HISEA-predicted natal origins of YOY match those of the DFA and the overall HISEA classification accuracy of post-settlement YOY is 98% (±2%) in 2008 and 96% (±4%) in 2009 (Table 3).

The second source of uncertainty results from the possibility that not all possible source populations were sampled. Given the extensive population surveys conducted prior to the study to identify possible source populations and considering the classification methods predicted the natal origin of YOY with high probability (i.e. >96%) we have high confidence in the predicted natal origins. However, the possibility does exist that an individual originated from an unsampled location outside of the study domain that happened to have an elemental fingerprint indistinguishable from one of our study sites, resulting in misclassification of its natal origin. If this did occur, there would be inaccuracies in the described population connectivity patterns, but without exhaustive sampling from all possible sites at all possible times, this claim cannot be refuted nor supported with data or samples in hand. However, we can use the estimated dispersal distances to place bounds on the probability that a YOY collected at the various study sites may have originated from an unsampled site outside of the study domain.

Assuming mean (±1 se) dispersal distance (excluding individuals predicted to have self-recruited) across both years of the study was consistent in regions adjacent to the study region and similarly ranged from a low of 5 km (in a southerly direction) to a maximum distance of 59 km (in a northerly direction), and knowing the nearest unsampled populations were ∼30 km to the south and 50 km to the north [28], we can estimate the proportion of YOY that may have originated from an unsampled location, but managed to disperse into the study domain. In a southerly direction the mean dispersal distance was 13.3 km (±3.9), with a range of 5.0–27 km among the two-week nesting bins across both years of the study. As the nearest known source population outside of the study domain lies ∼50 km north of the northernmost study site, this suggests no individuals invaded the study domain from the north to be misclassified. In a northerly direction the mean dispersal distance was 27.6 km (±1.6). This is the approximate distance from the southernmost study site (Zuniga Point) to the nearest known source population to the south of the study domain. Therefore there is a possibility that the single YOY collected at Zuniga Point in 2009 did not come from La Jolla as predicted, but rather an unknown unsampled source to the south of the study domain. For each individual two-week nesting bin across 2008 and 2009 the mean northward dispersal distance ranged from a low of 14 km (June 01–15, 2009) to a high of ∼46 km (July 16–31, 2009), and the longest distance a single individual dispersed in a northerly direction was 59 km. If we take this proportion (1/89) of YOY, and apply the same logic to individuals dispersing from an unknown unsampled site to the south of the study region, there is an approximately 1% chance that an individual developing on a benthic nest between July 16–31, 2009 may have dispersed from an unknown unsampled site to either La Jolla (57 km north of the nearest unsampled site) or Mission Point (43 km north of the nearest unsampled site). However, when the mean northerly dispersal distances of YOY and YOY capture data are explored further, during this same two week period of highest mean dispersal distance (July 16–31, 2009), five YOY were captured at Carlsbad (89 km from the nearest unsampled site) and Cardiff (76 km from the nearest unsampled site); during this period of time no YOY settled and were captured at the three southernmost study sites (Zuniga Point, Mission Point or La Jolla). Therefore, while we cannot say with 100% certainty that an individual has not invaded the study domain from an undocumented unsampled site to the south of the study region, we can say that based upon the empirical evidence, and given the constraints associated with the otolith microchemistry classification methods, we are confident that our natal classifications and resulting connectivity patterns are representative of actual patterns.

There is also the possibility of contamination in the mounting medium that could result in uncertainty regarding the veracity of our conclusions. Great efforts were taken as part of our methods development to test several mounting media, and identify and use the cleanest mounting medium possible. To minimize the possibility of contamination all otolith processing was conducted in a class 100 clean room, and samples stored within a class 100 laminar flow hood housed within a class 10 ultra-clean room within that larger class 100 clean room. The double-sided tape ultimately used (Scotch 3M Permanent Double Sided Tape, #665, ½”×500” (13.8 yds), 12.7 mm×12.7 mm, “Made in the USA”) was the “cleanest” mounting medium tested, and has been used in several other published studies (e.g. [19], [26], [35], [47], [48]). We used the same roll of tape for all samples analyzed, and several tape blanks were analyzed on every mounting slide to verify there was no contamination of samples.

To verify that our mounting tape was not contaminated with respect to any of our target trace elements (^24^Mg, ^48^Ca, ^55^Mn, ^87^Sr, ^138^Ba, ^208^Pb, and ^238^U; these elements were themselves vetted with trace elemental proof-of-concept data generated as part of a pilot project in 2007/2008), raw count data from the ICPMS was subjected to further statistical analyses. The mean raw counts from our tape blanks were significantly lower for all trace elements relative to otolith material and analytical solution blanks run prior to and following analyses of each sample slide. For example, mean counts per second for ^48^Ca for otolith material were 1,229,994; for solution-based analytical blanks were 13,845; and for tape blanks were 7,965. Our tape blanks were significantly “cleaner” than both the otolith material and the analytical blanks (One-way ANOVA F_2,1990_ = 229.4, p = 2.21×10^−90^). In Tukey HSD post-hoc tests our tape blanks were also significantly “cleaner” than the analytical blanks with respect to ^48^Ca (p = 9.17×10^−6^).

All mounting tape blanks fell below pre-determined detection limits (i.e. >3 standard deviations above background blanks, as described in the Methods section). However, for comparative purposes mean counts per second (cps) for tape blanks were calculated and as mentioned, all were significantly lower than counts per second for otolith material (One-way ANOVA p values for individual elements provided below). The mean cps values for all trace elements used to recreate connectivity patterns as part of this study were:

^24^Mg = mean cps = 193,069 (otolith) >4722 (tape), p = 1.56×10^−15^
^55^Mn = mean cps = 37,085 (otolith) >680 (tape), p = 1.98×10^−8^
^87^Sr = mean cps = 371,065 (otolith) >2157 (tape), p = 1.8×10^−30^
^138^Ba = mean cps = 135,738 (otolith) >1419 (tape),  =  p = 1.49×10^−12^
^208^Pb = mean cps = 5265 (otolith) >33 (tape), p = 4.05×10^−6^
^238^U = mean cps = 893 (otolith) >8 (tape), p = 3.18×10^−8^

These values provide evidence that the mounting tape was significantly lower in all target isotopes than in otolith material, and supports our contention that contamination from mounting tape was highly improbable. However to assure that there was no contamination owing to lead, we analyzed our data omitting lead. The ensuing connectivity patterns from these analyses (both DFA and HISEA; Figure 2, Figure 4, Table 3), are identical to the results generated from analyses including lead. With these additional supporting lines of evidence, we have greater confidence in our results and the accuracy of our recreated connectivity patterns.

### Population Connectivity

To date, empirical studies estimating the magnitude of regional connectivity among MPAs have provided only snapshots of connectivity (i.e. one or two estimates per year) (e.g. [22], [23], [26]). This uncertainty has made extrapolations beyond and interpolations between data sets tenuous, and limited the successful application of connectivity estimates to the design of MPA networks [49]. By documenting larval connectivity patterns at high frequencies along the San Diego coastline we reveal that *H. rubicundus* connectivity, while generally northward on annual time scales can, in the short-term, be highly variable in both direction and magnitude (e.g. Figure 4). Insular studies assessing self-recruitment and connectivity in MPAs have shown relatively high levels of self-recruitment and connectivity [22], [23]. However, the frequency of self-recruitment of fish along open coastlines has remained unsubstantiated, and until now empirical evidence of self-recruitment and/or connectivity among populations inhabiting MPAs located along an oceanographically dynamic open coastline have been few. This evidence can be used by decision-makers to create MPAs (e.g. in regions of higher self-recruitment), determine how far to space MPAs and where to allow resource extraction (e.g. in non-source populations), thus providing benefits to consumptive and non-consumptive users.

The La Jolla MPA is located approximately 25 km from the Cardiff and Zuniga Point MPAs, to the north and south respectively. When viewed in terms of the mean dispersal distances measured as part of this study (27.6±1.6 km), it suggests these three MPAs are well sited in terms of distance between reserves. In addition we observed three individuals dispersing ∼5 km in a southerly direction, from Torrey Pines to La Jolla, over the 20 day pelagic larval period. Therefore it is plausible that the primary La Jolla MPA is connected to rocky reefs located to the south. Also as the rocky coastline stretching along this subregion provides an almost continuous strip of nearshore high-relief rocky habitat (the preferred habitat of *H. rubicundus*), this region most probably functions as one large subpopulation, albeit with significantly lower densities once you move south of the La Jolla MPA (Table 5, Figure 1).

A question critical to understanding marine metapopulation dynamics is, how does larval swimming behavior influence larval dispersal trajectories and ensuing patterns of connectivity? While spawning occurred throughout the three-month spawning season, settlement of YOY was episodic (*sensu* [50]); YOY arrived at sites intermittently and in what could be described as settlement pulses. Additionally, when connectivity estimates were viewed as a function of adult population size, certain sites (e.g. La Jolla) contributed significantly more settlers to the greater metapopulation than would be expected by chance. A possible mechanistic explanation for these cohesive settlement pulses of YOY is larval swimming behavior and physical processes acting in concert to aggregate dispersing larval fish during the pelagic larval period. Ben-Tzvi et al. [17] used otolith microchemistry to infer *Neopomacentrus miryae* (a tropical damselfish) form cohorts that spend their pelagic larval periods dispersing together, but complementary molecular analyses showed these individuals while dispersing cohesively were not related. In a different study in French Polynesia it was shown that unlike *N. miryae*, settlers of *Dascyllus trimaculatus* (another tropical damselfish) were related despite protracted pelagic larval periods [18]. Whether this cohesive dispersal occurs in temperate fishes and the extent to which various processes, physical oceanographic and/or behavioral, contribute to this phenomena are open questions. This line of exploration about aggregating behavior in larval fish has important ramifications for marine population dynamics and warrants further study.

Examination of empirically estimated mean dispersal velocities with concurrent ADCP data allowed a *post hoc* comparison of microchemistry-derived dispersal estimates with Lagrangian-based predictions of a passively dispersing particle (i.e. a proxy for a dispersing larval fish). Not surprisingly these physical data suggest a dispersing particle would travel very different distances depending on its vertical location within the water column. ADCP data suggest that if larval fish spend their entire 20-day pelagic larval period dispersing in the upper 2 m of the water column, they would be blown onshore due to prevailing winds. However, if larval fish spend those same 20 days below 4 m water depth they would be predicted to, on average, disperse ∼35 km alongshore. If we assume larval fish maintain their position within a given depth bin with no vertical movement into other depth bins (a large assumption), the range of dispersal velocities generated by empirical data from this study suggest larval *H. rubicundus* disperse in the mid to lower portions of the water column. As no vertical distribution data are available, studies documenting *in situ* larval vertical position and swimming behavior over a protracted dispersal period will be critical to validating this hypothesis.

Lab-based experimental studies of larval swimming behavior suggest late-stage larval damselfish can swim against a 13.5 cms^−1^ current between 25 and 250 hours, equivalent to swimming between 12 and 123 km [51]. Also swimming ability increases with ontogeny; at hatch *Pomacentrus amboinensis* (a tropical damselfish) can sustain swimming at 3.5 cms^−1^ for minutes, but by day 20 they can swim at >30 cms^−1^ for >90 hours [52]. *In situ* studies while few, suggest larval fish are cognizant of swimming directionality, and can maintain swimming speeds of >13 cms^−1^ [53]. Qualitative evidence suggests *H. rubicundus*, as with other pomacentrids, are active swimmers upon hatch [28]. Dispersing individuals may maintain their alongshore position in the water column by moving to portions of the water column where current velocities are lower, moving vertically to take advantage of currents flowing in opposite directions, swimming against currents, or some combination thereof. To further our understanding of the processes ultimately responsible for the breadth of dispersal trajectories and connectivity patterns exhibited by *H. rubicundus* will require further study pairing the collection of physical oceanographic data concurrently with pertinent biological data (e.g. vertical swimming behavior). Our understanding of population connectivity will benefit greatly from the development of integrative methods for exploring how variability and ontogenetic shifts in behavior ultimately impact metapopulation dynamics [10].

In 2008, eight individuals dispersed northward from La Jolla to Cardiff, and in 2009 two individuals dispersed in a southerly direction to re-connect the Cardiff population with the La Jolla population. Closing the connectivity loop between La Jolla and Cardiff has important implications for the persistence of populations inhabiting these MPAs; returning home (*sensu* [54]) is critical to the long-term persistence of marine metapopulations. The variability in connectivity patterns observed in this study has important implications for the design and management of MPAs; if connectivity patterns are viewed over short temporal scales, data incorrectly suggest this six-reef network functions as a source-sink metapopulation. However, when connectivity patterns are summed over inter-annual time scales, the metapopulation is more aptly described as a well-mixed metapopulation. A recent population connectivity study spanning an open coastline in the northeast Atlantic found site fidelity to adult fishery grounds in *Sardina pilchardus* populations. However the majority of adults appeared to originate from a single northern recruitment area, suggesting sardine populations along the Iberian Peninsula function as a classic metapopulation [45], [55]. The ability to distinguish between various metapopulation models results ultimately in better-informed management decisions.

In this study three MPAs within a larger network of reefs were connected by larval dispersal over a two-year period. However the predominant direction and strength of this connectivity varied. By assessing larval dispersal over various temporal scales, we show high-resolution sampling is critical to the accurate re-construction of population connectivity patterns. To fully-resolve connectivity patterns requires protracted time-series and synoptic sampling, particularly as self-recruitment and connectivity are central tenets for the success of MPA networks. In this study there appears to be sufficiently high self-recruitment within the La Jolla MPA that, taken in conjunction with the mean life span of the model species (∼13 years), this population may persist without larval inputs from outside the study region. Despite the high current velocities measured, larval dispersal ranged between 0 km and 59 km over a three-week period, further suggesting the importance of larval swimming behavior and/or physical retention mechanisms to realized patterns of reef connectivity. In sum, the findings of this study lend support to the growing body of evidence for limited larval dispersal and self-recruitment, document the high frequency of directional shifts in connectivity patterns, and highlight the ramifications of this variability for future studies of marine metapopulations along oceanographically complex open coastlines.
